# “Having the compass–drawing the map”: Exploring nurses’ management of pain and other discomforts during use of analgosedation in intensive care

**DOI:** 10.1002/nop2.227

**Published:** 2018-12-18

**Authors:** Helene Berntzen, Ida Torunn Bjørk, Hilde Wøien

**Affiliations:** ^1^ Department of Postoperative and Intensive Care, Division of Emergencies and Critical Care Oslo University Hospital Oslo Norway; ^2^ Department of Nursing Science University of Oslo Oslo Norway

**Keywords:** analgosedation, clinical decision‐making, critical care, nursing process, observation, pain management

## Abstract

**Aim:**

To explore the deliberation and enactment processes of nurses in relation to pain and other discomforts in the critically ill patients after the implementation of an analgosedation protocol.

**Background:**

Nurses in intensive care units (ICU) face great challenges when managing pain and other discomforts and distinguishing between patients’ needs for analgesics and sedatives. An analgosedation protocol favouring pain management, light sedation and early mobilization was implemented in a university hospital ICU in Norway in 2014. Changing sedation paradigms resulting in an increasing number of awake patients during critical illness is expected to affect nursing practice.

**Design:**

Exploratory, single‐unit study in a mixed adult ICU.

**Methods:**

Data collection with participant observation and semi‐structured interviews in sixteen clinical situations in 2014 and 2015. Thirteen experienced certified critical care nurses were included. Thematic content analysis was conducted.

**Results:**

An overall theme “*Having the compass–drawing the map*” emerged from the analysis. The protocol or strategy of analgosedation appeared to provide a direction for treatment and care, although requiring extensive interpretation of needs and individualization of care, often in challenging situations. The overall theme was abstracted from three themes: *“Interpreting a complex whole,” “Balancing conflicting goals” and “Experiencing strain from acting across ideals.”*

**Conclusion:**

Nurses seem to attend adequately to patient pain, but the approach to discomforts other than pain appears unsystematic and haphazard. More explicit goals of care and strategies to handle discomfort as distinct from pain are needed. More research is needed to identify effective comfort measures for ICU patients.

## 1 INTRODUCTION

Clinical decision‐making is a principal nursing skill and is highly complex in the context of critical care (Aitken, Marshall, Elliott, & McKinley, 2009). According to Bucknall (2000), nurses in intensive care units (ICU) face a decision or judgement every 30 s. Nurse decision‐making in the ICU related to pain and other discomforts often relies on variables other than self‐reporting. The patient's ability to communicate needs is often impaired due to critical illness, sedation, mechanical ventilation (MV) and cognitive impairment. Deep sedation in ICU patients has been associated with poor long‐term outcomes for mortality and psychological recovery (Shehabi et al., 2012). International recommendations of light sedation (Barr et al., 2013; Strøm, Martinussen, & Toft, 2010) aim to contribute to more awake patients able to communicate pain and other needs. Many patients, however, suffer from procedural pain (Puntillo et al., 2014) and other discomforts (Berntzen, Bjørk, & Wøien, 2018). Unrelieved, pain may have serious physiological and psychological consequences (Jones et al., 2007; Sessler, 2009).

Analgosedation as a strategy complies with current recommendations of light sedation by aiming at assessing and treating pain first and providing sedatives only when necessary to help patients to rest and to reduce anxiety and agitation (Devabhakthuni, Armahizer, Dasta, & Kane‐Gill, 2012). Valid assessment tools are crucial in making appropriate decisions about pain in ICU patients unable to communicate (Barr et al., 2013; Gelinas & Johnston, 2007). Indicators of pain in such tools are facial expression, body movements, muscle tone and ventilator compliance (ibid).

This study aimed to explore the management of patient pain and other discomforts by ICU nurses after implementation of an analgosedation protocol.

### 1.1 Background

Decision‐making by ICU nurses is complex because the patients are seriously ill and their health status changes rapidly (Bucknall, 2000, 2003), often requiring nurses simultanously to deal with aspects of assessment, physiology and treatment (Aitken et al., 2009). Clinical decisions are influenced by the nurse's individual knowledge and experience (Bucknall, 2000, 2003; Shannon & Bucknall, 2003). Lack of knowledge and inappropriate assessment procedures partly explain the continuing reports of under‐treatment of pain and over‐sedation in ICU patients (Gelinas, 2016; Pasero et al., 2009). The importance of systematic assessment of pain and sedation with validated tools has therefore been emphasized (Barr et al., 2013; Payen et al., 2007; Wøien, Værøy, Aamodt, & Bjørk, 2012).

Pain is experienced by medical, surgical and trauma patients at rest and during medical and nursing procedures and is considered a great source of stress (Barr et al., 2013). A wide range of other discomforts also identified as distress or stressful experiences are also reported, including delusions, anxiety, immobility, inadequate sleep and communication problems, frequently related to MV (Berntzen et al., 2018; Karlsson, Lindahl, & Bergbom, 2012; Samuelson, 2011; Stein‐Parbury & McKinley, 2000; van de Leur et al., 2004). In this study, we define discomfort according to Kolcaba, as an umbrella term including pain (Kolcaba 2003, www.thecomfortline.com).

According to Vincent et al (2016), a multi‐professional approach to patient comfort in ICU is needed. Vincent and intensive medicine fellow researchers claim that the main goal is a comfortable, calm and cooperative patient, able to engage with family and caregivers. To achieve this, analgosedation should be provided and care should be humane and person‐centred to ensure a health‐promoting environment (ibid). It is paramount that nursing practice in ICU continues to reflect these recommendations.

Protocols may assist nurses and other healthcare professionals (HCP) in making decisions, also with regard to provision of analgesia and sedatives (Brattebø et al., 2002; Brook et al., 1999; Minhas, Velasquez, Kaul, Salinas, & Celi, 2015). However, low adherence to protocols and guideline recommendations (Mehta, McCullagh, & Burry, 2009; Rycroft‐Malone, Fontenla, Seers, & Bick, 2009; Sneyers, Laterre, Perreault, Wouters, & Spinewine, 2014) has been related to concerns about patient comfort and safety when treated with no sedation or light sedation (Rose et al., 2015; Sneyers et al., 2014) and concern that protocols might hinder clinical judgement (Wøien & Bjørk, 2013).

Customizing strategies when implementing recommended practices requires an understanding of practice patterns and beliefs (Rose et al, 2015). To understand and improve care and thus enhance comfort for critically ill patients, nurses need a comprehensive understanding of the clinical issues that contribute to pain and discomfort and how contextual factors influence decision‐making regarding comfort. Thompson, Aitken, Doran, and Dowding (2013) emphasize the need to access the logic behind decisions to be able to “unpack the quality of a choice” (p. 1,722).

**Figure 1:**
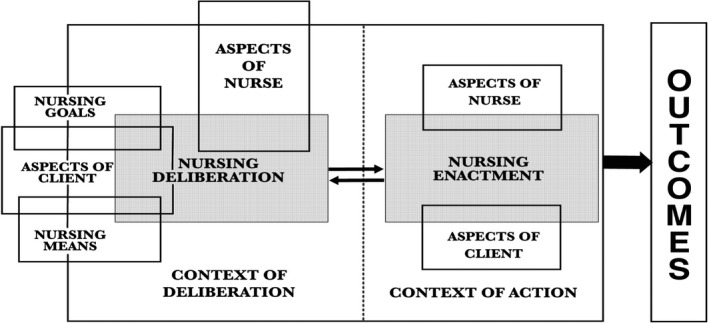
The processes of deliberation and enactment (Kim, 2010). (Reprinted with permission)

In Kim's framework of nursing practice (Kim, 2010), the terms “deliberation” and “enactment” describe distinct processes in nursing practice. Deliberation involves the process of clinical decision‐making including structuring of information, judgement about the meaning of the information and arriving at decisions on how to act. Enactment describes the nursing intervention or action. In the framework, the two basic processes encompass a complex series of actions involving different structural units (Figure 1). The processes are not linear and sometimes overlap, but they may be analytically separated for the purpose of understanding nurses’ clinical practice (Kim, 2010).

Kim's framework and concepts seemed useful in exploring and describing nurses’ involvement in clinical situations.

According to Kim, nursing science should seek to obtain knowledge to increase “the proportion of rational and explained acts in the total repertoire of what the nurse does in nursing” (Kim, 2010, p. 191).

## 2 THE STUDY

### 2.1 Aim

The aim of this study was to explore the characteristics of deliberation and enactment by ICU nurses in relation to pain and other discomforts in critically ill patients after the implementation of an analgosedation protocol.

### 2.2 Design

This study was part of a larger implementation study with the overall aim to follow the progression of pain, agitation and delirium (PAD)—practice after implementation of an analgosedation approach. An exploratory design with naturalistic observation and semi‐structured interviews was used. Naturalistic observation provides data on phenomena difficult to understand as it gives access both to what people do and what they say they do (Green & Thorogood, 2013). This method can provide a more complete understanding of the complexities of a situation.

### 2.3 Setting and participants

The study was conducted in a Norwegian university hospital. The characteristics of the setting are provided in Table 1. Patients treated in this study were aged 18–78 years and had various diagnoses, predominantly cardiorespiratory complications. Some were awake and some deeply sedated, in general with low pain scores.

Criterion sampling was used (Patton, 2002). Certified critical care nurses working permanently in the study unit 50% or more and for a minimum of two years were recruited. Inclusion criteria of employment and experience were used to ensure a reflection of the practice in the study unit where the analgosedation protocol had been implemented. An invitation to participate was distributed to all eligible nurses (approximately 80). Twenty‐five female and two male nurses consented by returning a reply form. Thirteen of these, all female nurses, were then consecutively included for participation, on shifts where observation was scheduled and the nurses were assigned to patients who had consented. After 16 observations, data were considered sufficient to secure *information power* (Malterud, Siersma, & Guassora, 2015). According to Malterud, saturation of data is not a realistic goal in an exploratory study, but the design of the study allowed for continued inclusion if the data generated were not considered sufficient to meet the aim of the study.

**Table 1:** Setting

ICU	11‐bed mixed adult ICU Single rooms and rooms with three beds separated by curtains
Staffing	Nurse:patient ratio 1:1 An extra nurse was available when required
The analgosedation protocol implemented before this study	Assessment of pain, agitation, sedation and confusion with valid tools at least every 8 hr or once per shift, Numeric Rating Scale (NRS) for pain assessment for patients able to self‐report and Critical‐Care Pain Observation Tool (CPOT) for patients unable to self‐report Richmond Agitation‐Sedation Scale (RASS) for agitation and sedation. Confusion Assessment Method (CAM‐ICU) for confusion and delirium Analgesia provision first, sedation only when necessary Suggestions for a range of pharmacological treatments for different patient categories Short‐acting medication for procedures Advise early mobilization
	

### 2.4 Data collection

Data were collected between November 2014–June 2015 in 16 situations using participant observation and semi‐structured interviews (Green & Thorogood, 2013; Hammersley & Atkinson, 2007) involving 13 nurses and 12 patients. Three nurses were observed twice and two patients were cared for several times, but situations never included the same nurse and patient dyad. All data were collected by the same experienced interviewer. She was an experienced ICU nurse who had no familiarity at the study unit. In the present study, we were not explicitly aiming to explore the negative or positive implications of using the analgosedation protocol and therefore the protocol itself was not a main concern during interviews and observations. We were open‐minded to how and to what extent the protocol was applied without putting an emphasis on the use, thus avoiding to normatively influence on how the nurses practiced or talked about the management of pain and other discomforts.

#### 2.4.1 Observations

The nurses were observed for 60–150 min (mean 110). An observation guide was used. Observations focused on activities like handover and shift reports, initial assessment of patient and planned procedures possibly requiring deliberations or enactments regarding pain or other discomforts. Non‐pharmacological and pharmacological responses to patient cues of pain and other discomforts and adherence to the implemented protocol were specifically observed. Nurses were encouraged to care for patients as usual, but to think aloud when relevant regarding pain and other discomforts. Short field notes were taken during observation, especially to capture any occurring dialogues. Extensive notes were written after each observation.

An “observer as participant” approach was adopted (Hammersley & Atkinson, 2007), implying that observation was the primary activity, yet involving some participation or interaction. Informal questions and small‐talk conversations were used to enhance trust and promote rich data. The observer wore private clothes, a white coat and a research‐nurse badge to blend in, but to avoid being mistaken for a nurse at work.

#### 2.4.2 Interviews

Semi‐structured interviews lasting 11–34 min (mean 21) were conducted as reflective dialogues (Graneheim, Lindgren, & Lundman, 2017), focusing on clarifying observed behaviours and investigating deliberations and enactments regarding patient pain and other discomforts. All interviews were conducted during the observation shift and in all but two cases, field notes were written before the interview to reflect on the questions to ask. A short interview guide supplemented these questions (Table 2) and opening questions like: “What are your thoughts about the pain and discomfort of your patient on this shift?” were posed to encourage narration. The implemented protocol was not specifically discussed, unless initiated by the nurse. Interviews were recorded and transcribed verbatim by the interviewer.

**Table 2:** The interview guide

Introduction What are your thoughts about pain and discomfort of your patient on this shift?
Assessment Can you tell me about how you assess pain and other discomforts? What would you say influences how you assess?
Interventions What are your goals when intervening towards pain and other discomforts? Can you tell me about any interventions you made today? What would you say influences how you intervene?
General Prompting questions Clarifications/elaborations specifically related to the observations?

#### 2.4.3 Pilot study

Two pilot observations and interviews were performed to test and elaborate the guides and to familiarize with the data collection method. The guides, however, were continuously developed throughout the study, to sharpen the focus on the process of deliberation and enactment.

### 2.5 Ethical considerations

The study was approved by the regional committee for medical research ethics (Health Region East, Norway; Project –ID; 2014/125) and conducted according to the Declaration of Helsinki (WMA, 2013). Written and oral information was provided and written informed consent was obtained from participating nurses and from patients enrolled in the study (or from their closest relatives).

### 2.6 Data analysis

A thematic content analysis was conducted (Green & Thorogood, 2013). Data were coded inductively, but Kim's framework of nursing processes was used as a scaffold, to assist in searching for patterns, similarities and inconsistencies in nurses’ deliberation and enactment. NVivo Version 11 (QSR International Pty Ltd., 2015) was used to organize data. As we considered that the observations contained the core data, two researchers individually read the field notes several times to familiarize with the data and to obtain an overall impression (Green & Thorogood, 2013). The second step involved identification of initial codes encompassing enactments related to the research questions. Five initial codes were agreed on through elaboration and discussion (Table 3). The first three codes came naturally as a consequence of data obtained using the observational guide and the latter two emerged from data in the reflective field notes. Six of the interview transcripts were then read thoroughly to identify deliberations connected to each enactment code. For each initial code, groups of codes with similar content were established and a coding scheme was created. The third step involved coding of the remaining interviews in a dynamic process to allow identification of new codes, rearrangements and creation of new code‐groups. The final step, comparing and contrasting the code‐groups across the whole data set, resulted in seven categories. Three abstracted themes were identified through exploration of the relationship between all categories. A final abstraction constituted the overall theme “*Having the compass–drawing the map*.” Table 3 illustrates the process of analysis showing the steps from initial codes to the overall theme.

**Table 3:** The analytical process showing the initial codes, code‐groups, categories and themes (Green & Thorogood 2013)

Initial codes	Code‐groups	Categories	Themes	Overall theme
Assessing pain and other discomforts	Using valid tools Challenges and barriers to assessment Schedule of assessment Assessing other measures not captured by tools	Facilitating tools, but still requiring interpretation Collecting and combining cues during routines and continuous care Enacting on information from different sources	Interpreting a complex whole	Having the compass–drawing the map
Combining information	Using available information Interpreting Attending to patient preferences			
Choosing interventions directed towards pain or other discomforts	Adhering to principles of protocol Using pharmacological interventions Applying non‐pharmacological interventions Choosing between pharmacological and non‐pharmacological interventions	Ensuring aspects of pain relief and comfort Ensuring the aspect of rehabilitation	Balancing conflicting goals	
Handling other treatment goals along with comfort	Achieving a good balance between goals Experiencing a difficult balance between goals Prioritizing between goals of comfort and pain relief and of rehabilitation Using professional repertoire Using personal repertoire			
The cost of professional and personal involvement	Acting at variance with professional conviction Acting at variance with personal standards Observing and withstanding patient discomfort and suffering	Experiencing threats to professional ideals Experiencing threats to personal standards	Experiencing strain from acting across ideals	

This analytical approach enabled the elucidation of how ICU nurses think and what they do, from patterns across all 16 observations and interviews. In this way, the presentation of findings involves a holistic description of the characteristics of the processes of deliberations and enactments regarding pain and other discomforts when analgosedation is used.

### 2.7 Rigour

Lincoln and Guba's framework is used to describe trustworthiness (Lincoln & Guba, 1985). Triangulation of research methods was used to obtain in‐depth data and enhance *credibility*. *Dependability* was sought by describing the role of the researcher and the data collection details. All analytical steps were performed by more than one researcher to ensure different perspectives and to enhance *confirmability*. The process of analysis was described and displayed in a table, and the findings were discussed with ICU nurses to confirm recognition of categories and themes. *Transferability* to similar contexts was sought through descriptions of the setting and the participants and by rich descriptions of the findings, accompanied by illustrative quotations.

## 3 FINDINGS

The study participants were aged 38–59 and had been working in ICU for an average of 19 years. The overall theme “*Having the compass–drawing the map*” indicates that the implemented strategy of analgosedation provided a direction for treatment and care, although it was seldom explicitly referred to or discussed by the nurses during observations or interviews. Extensive interpretation of patient needs and individualization of care was still required, often in complex situations. The overall theme was abstracted from the following themes: “*Interpreting a complex whole,” “Balancing conflicting goals” and “Experiencing strain from acting across ideals*” (Table 3).

### 3.1 Interpreting a complex whole

In spite of the strategy implemented, the nurses were required to collect and combine cues and make interpretations to make decisions and act. Valid assessment tools for pain and sedation levels were widely used and perceived to facilitate a systematic approach to assessment, reduce subjectivity and enhance more consistent reporting. Often elements from tools were incorporated into continuous assessment. Usability was also reflected in comments indicating that tools were just a new wrapping:

The way the CPOT is built, it is actually based on things we used to assess from before, but now exactly what to do and what points to give is more specified. (..) And earlier, even though you would observe facial expression and cooperation with the ventilator, it was more open to individual interpretation. (interview 1)

Despite the availability and usability of valid tools, sedation and impaired communication and cognition often required interpretation beyond scoring. Sometimes messages were conflicting regarding patient self‐reporting of pain and the patient behaviour observed. Other times the scoring alternatives seemed too confining to cover the level of pain or sedation and to reach a common understanding:

Nurse: “How bad is your pain right now?” Patient’s eyes are closed. She hesitates…”ehm..3…” Nurse; “But can you perhaps live with it?” Patient: “But it really hurts.” Nurse: “but 3 isn’t that much on the scale…maybe it is more?” Patient: “4?” (with a questioning tone). (field note 6)

In general, it was not clear how the nurses deliberated to distinguish between pain and other discomforts and no systematic assessment of discomforts other than pain was evident. However, one way of describing discomfort was mild pain or a precursor of pain requiring an enactment. There was general agreement that no patient should be in pain, but that not all discomfort could be eliminated. However, when nurses were unable to decide on what was pain and what was discomfort, the assessment and the decision about when and how to intervene were challenging.

Nurses discuss patient distress at handover

‐ But it is agitation, not pain?

‐ Who knows? I don't know why she was crying, I can't get eye‐contact because she's too sedated in a way, but at the same time in distress. (field note 16)

To know the patient well added depth to the deliberation but did not necessarily facilitate the decision about what to enact. This nurse had been caring for a young man with cancer and septicemia even before he was sedated and intubated:

…and when he has expressed a wish to die ‐ and believes he is going to die, what is agony, what is discomfort, what is pain? I mean what is what? It is really difficult. (interview 2)

The nurses assessed pain as part of a daily routine, such as when starting the shift, during daily care and often specifically when turning the patient. The assessment included combining scorings and other measures or input not captured by the tools. Vital signs and other physiological measures like lacrimation and restlessness were used along with information from patient records and from handover and inter‐professional rounds. Patient history, current medication and the reporting nurse's perceptions contributed to interpreting the pain situation in patients who were unable to self‐report. Frequently nurses deliberately performed turning or repositioning without any pre‐emptive or extra medication “to obtain a better impression” of the pain and sedation level:

I thought I had to …ehm .. allow myself to see how he actually is – and how it turns out before I decide in a way, because at first I thought I had to give both ketorax and propofol beforehand, but if I give it before I start, I don’t really know anything about him. (interview 2)

Several nurses described the demanding situation of having more awake patients on MV requiring constant attention and surveillance, frequently disrupting workflow. However, most emphasized the importance of being guided by patients’ responses in making decisions about pain and other discomforts. The nurses felt safer doing the right thing when patients could respond:

The patient is clearly grimacing when they turn her over. The nurse asks whether she is in pain. She frowns and shakes her head. “Discomfort?” the nurse asks. The patient nods. The nurses discuss whether to give more analgesia or propofol (sedative). The nurse turns to the patient and asks whether she just wants something to make her relax. She nods again and they give her 10 mg propofol. (field note 4)

### 3.2 Balancing conflicting goals

The second theme reflects how the nurses needed to prioritize between different and sometimes conflicting goals of relieving pain and other discomforts, yet ensuring goals of progress and rehabilitation. An effort to outweigh the pain and discomfort was made by use of pharmacological and non‐pharmacological interventions and evident in both observations and interviews. Pharmacological treatment was mainly directed towards possibly painful procedures like endotracheal suctioning, repositioning and mobilization in or out of bed:

I feel very anxious about my patients being in pain, so the pharmacological options are often the first priority. (interview 8)

Even though the previously implemented analgosedation protocol itself was seldom mentioned, the nurses gave a general impression of good understanding and adherence to the principles of analgosedation as a general direction for treatment and care. There was, however, a concern about how the goal of keeping patients more lightly sedated or awake was experienced by the patients:

The patients are supposed to be more awake …even though it may result in a bad experience (..) it’s for better or worse I think….but I hope we have sufficient tools to capture whether they can tolerate it. (interview 11)

Nurses acknowledged to a large extent the non‐acute pain and discomfort resulting from being critically ill, insecure and frightened, immobilized and awake. A caring approach using continuous information, therapeutic touch and soothing speech was observable as the enactment in caring for patients who were sedated. A personal repertoire of skills was observed in balancing the aspect of comfort and the rehabilitative aspect when patients were more awake. The skills encompassed creativity, humour, motivational skills, “standing by” and recognition of the individual:

When you have someone awake like her, you can't keep going at 110, …you need to slow down and work at a different pace…(interview 7)

The overall goals of rehabilitation sometimes caused the nurses to withhold analgesics or sedatives to accomplish planned or prescribed events such as waking patients who had been sedated, weaning from the ventilator, maintaining spontaneous breathing or mobilizing in or out of bed:

It is this knife‐edge we are balancing on – when I provide too much analgesics, I get a sedated patient who is not coughing and who is not moving and who does not communicate. (interview 6)

Other times “balancing” implied cancelling physiotherapy and mobilization and letting the patient rest to recover. Such a balance was not always achieved and the nurses sometimes had to handle challenging situations across their ideals of care.

### 3.3 Experiencing strain from acting across ideals

Nurses were affected by witnessing and withstanding patient discomfort and suffering and by sometimes having to disregard their professional ideals and personal standards to balance the comfort and rehabilitation of patients.

The strain experienced by the nurses seemed linked to their professional and personal involvement. To abide strictly by professional standards might challenge the nurse's personal standards of care. Despite being given both analgesia and tranquillizers, a woman cared for in one situation continuously expressed pain verbally. Because of her reduced respiratory function, withholding intravenous opioids was prescribed:

Nurse: “The case is that you got pain medication a while ago and it is important that you don’t get too sleepy”…. she goes on telling the patient that she is aware that she is being pushy, which is difficult because she needs to act according to her heart – not to be a torturer……”but we will work our way through this together – You and I!”. (field note 6)

Personal involvement with the patient situation, the history of the patient and the relatives seemed to contribute to vulnerability towards the suffering of a patient. However, the experience and knowledge of specific situations helped nurses withstand better patient discomfort and suffering:

You need a balance to get to an extubation for instance … like if we can manage this short, but steep hill, we can extubate and then things will be much better – then I think I can put up with a bit more. (interview 1)

Working towards common goals together with the patients when possible, and involvement of fellow nurses and physicians in their deliberations supported the nurses in their enactments.

## 4 DISCUSSION

A central finding in this study was that analgosedation principles were clearly visible in the management of pain and other discomforts in critically ill patients, although the ICU nurses involved seldom referred to the implemented protocol. This finding may indicate that the analgosedation principles are easily adopted and merge well with current clinical practice. We also found that the elements of the pain assessment tools used formed a “natural part” of the nurses’ routine assessments. Although interpretation beyond the tools was needed, there was fair agreement on how to assess and treat pain and that no patient should be in pain. Discomfort, however—although agreed on by the nurses as something that could not be fully eliminated in critically ill patients—seemed ill defined and difficult to distinguish from pain, in line with the findings of Gerber, Thevoz, and Ramelet (2015). The challenge of distinguishing between pain and discomfort in ICU patients also shown in our study complicate nurses’ deliberations and enactments. Berntzen et al. (2018) explored the experience of pain and other discomforts as separate entities in ICU patients treated with analgosedation. They found that the patients’ pain was largely relieved, but that they struggled with other discomforts, mainly due to MV, incomprehension and experiencing delusions. Lærkner, Egerod, Olesen, and Hansen (2017) found that being awake during critical illness increased the ICU patient's awareness of the severity of their illness and increased their discomfort and sense of incapacity.

Despite constantly considering discomforts like pain, anxiety, incomprehension, immobilization, constipation and equipment attached, nurses in our study seemed to lack systematic deliberations about discomforts other than pain. However, there was a tendency to consider discomfort as mild pain or as a precursor to pain, precipitating pharmacological interventions, mainly increasing of the opioid infusion rate or bolus injections. Non‐pharmacological interventions were applied to prevent or relieve both pain and other discomforts, but no systematic deliberation preceding the enactments was observable or articulated. Sometimes the nurse's deliberation about sources of discomfort was communicated at handover. In Kim's (2010) structure for nursing deliberation, the availability of nursing means is divided into “repertoire at large” which mainly applies to validated strategies and into “personal repertoire” or “conjectured means or approaches” (p. 186) related to the individual nurse. Nurses in this study approached pain through deliberation using valid assessment tools and adhering to treatment principles from a protocol, a practice corresponding to Kim's “repertoire at large.” The approach to both deliberating and enacting for discomforts other than pain, however, seemed highly based on individual or personal comprehension or interpretation of the patient situation, corresponding to Kim's “personal repertoire.”.

In a focus group study, although ICU nurses recognized the usefulness of pain and sedation assessment tools, they relied more on their personal knowledge and experience (Wøien & Bjørk, 2013). Clinical decision‐making based primarily on the personal repertoire and conjectures based on individual professional and personal standards reflect a haphazard or intuitive rather than an intentional practice (Kim, 2010). In a normative perspective, nurses should aim at delivering competent, timely, relevant and efficient nursing measures according to prescriptions or strategies (Kim, 2010). Considering the extent to which ICU patients experience discomforts other than pain (Berntzen et al., 2018), it seems necessary to aim at a more structured process of both deliberation and enactment to enhance patient comfort.

Structured processes need explicit goals. According to Kim, a set of goals can be identified in every situation involving nursing actions (2010), but one problematic aspect of the deliberation process is how the nurse perceives and prioritizes the different goals. Patient comfort in our study seemed to constitute an implicit goal of care (GOC). According to Stanek (2017), GOCs are ideally established and made explicit through interaction between a patient and healthcare professionals, but are frequently taken for granted and not explicitly articulated. A critically ill patient's ability to express comfort needs may be impaired, hindering the establishment of explicit goals of comfort. Kim divides the orientation of nursing goals into those defined by the client, by the nurse and by others. In our study, the nurses appreciated the support in decision‐making obtained through the awake patient's ability to express their needs. The nursing goals and hence means were then tailored to the patient's own preferences or goals. Juxtaposing elements of nursing means and nursing goals in the process of deliberation is necessary to make nursing practice coherent, meaningful, strategically effective and sensible (Kim, 2010, p. 187).

Over the past few years, strategies and bundles of treatment and care, representing evidence‐based guidance for clinicians, have been introduced and implemented in many ICUs. Focusing on comfort, the e‐CASH concept (early Comfort using Analgesia, minimal sedatives and maximal humane care) pursues the goal of a calm, cooperative and comfortable patient (Vincent et al., 2016). These goals of caring for critically ill patients in ICUs need to be operationalized to have the potential to help nurses structure deliberation and subsequent enactment, also for discomforts other than pain. Puntillo et al (2010) assessed ICU patients at a high risk of dying with a 10‐item checklist for the presence and intensity of symptoms and concluded that symptom assessment could lead to more focused interventions and reduce patient suffering. Chanques et al suggested the daily evaluation of five common stressful symptoms in ICU patients able to communicate: pain, thirst, anxiety, dyspnoea and poor sleep (Chanques, Nelson, & Puntillo, 2015). In a review of existing instruments to assess patient comfort during hospitalization, Lorente et al found moderate methodological quality and low reported utility of the tools (Lorente, Losilla, & Vives, 2017). None of these instruments, however, were developed specifically for the critical care context.

A systematic approach with strategies or tools may aid nurses in assessing and handling discomfort as distinct from pain. The lack of sensitivity in current tools may result in overestimation of pain and underestimation of other discomforts. Analgosedation as a strategy promotes pain assessment and treatment first and will enable more patients to express their needs. We need to ensure, however, that other discomforts are not mistaken for pain and treated as such in patients unable to communicate. Despite the need for tools and strategies to avoid haphazard deliberations and enactments, our study shows that nursing practice in intensive care is inevitably dependent on the nurses’ personal and professional engagement, skills and standards.

### 4.1 Limitations

There are several limitations to this study. Firstly, the single‐unit study design may reflect a local culture in the practice of decision‐making and therefore reduce transferability to other units. The nurse:patient ratio is considered high in Nordic ICUs (Egerod, Albarran, Ring, & Blackwood, 2013) and workload issues may influence the deliberations and enactments of nurses. However, no other healthcare staff or therapists are involved in the direct bedside care of the patient. Secondly, all the participants were female and a mixed sample might have revealed gender differences in decision‐making. However, most nurses in general and at the study site are female and comparison between genders was not part of the study aim. Thirdly, the data collector's familiarity with the field may have impaired the objectivity of the observations and analysis. Attempting to consciously balance between closeness to the field and the nurses, while focusing on the nature of the nurses’ deliberations and enactments, hopefully provided what is described as a creative insight using both an “insider” and “outsider” perspective (Hammersley & Atkinson, 2007). The collaboration of three researchers in the analysis, one without any relation to ICU nursing, may have counteracted the danger of a skewed analysis based on the preconceptions of the experienced ICU nurse collecting the data.

## 5 CONCLUSION

In this study, the use of Kim's framework to guide the analysis contributed to highlighting the differences between the structure of deliberations and enactments with regard to pain and other discomforts in ICU nursing practice. We showed that the processes of deliberation and enactment regarding pain adopted by the nurses, to a large extent relate to existing recommendations for preventing, assessing and treating pain. Protocols or strategies, reflected in our study by the analgosedation protocol, may be regarded as a compass to indicate the direction of treatment and care in nurses’ decision‐making. The “compass” adds to the metaphor “the landscape of critical care” used by Bucknall (2003) in exploring influences on nurse decision‐making. The valid tools, goals of pain treatment, guidelines and prescriptions may represent landmarks for nurses to find the “path.” However, the complex landscape of the critical care context will always compel nurses to use both personal and professional skills to navigate. Concerning discomforts other than pain, the landmarks to guide nursing practice are sparse. This results in extensive individual interpretation and judgement in nursing deliberations and enactments, as to whether or when to apply pharmacological or non‐pharmacological measures, often in situations lacking explicit or established goals. To avoid haphazard clinical practice in the ICU, new assessment methods that capture discomforts other than pain should be developed and effective comfort measures to relieve discomforts other than pain should be identified and implemented. Knowledge about ICU patients’ discomforts other than pain should be used to describe more explicit goals of care and ensure patient comfort.

## CONFLICT OF INTEREST

All authors declare no conflict of interest.

## AUTHOR CONTRIBUTIONS

HB, ITB, HW: Study design, analysis and manuscript preparation. HB: data collection.
